# A molecular classification system for estimating radiotherapy response and anticancer immunity for individual breast cancer patients

**DOI:** 10.3389/fonc.2023.1288698

**Published:** 2023-10-20

**Authors:** Jiaxuan Zhang, Long Li, Haotian Shang, Zhaoyan Feng, Tengfei Chao

**Affiliations:** ^1^ Department of Radiology, Tongji Hospital, Tongji Medical College, Huazhong University of Science and Technology, Wuhan, Hubei, China; ^2^ Department of Oncology, Tongji Hospital, Tongji Medical College, Huazhong University of Science and Technology, Wuhan, Hubei, China

**Keywords:** breast cancer, molecular classification system, radiotherapy, immunotherapy, chemotherapy

## Abstract

**Objective:**

Radiotherapy is a cornerstone of breast cancer therapy, but radiotherapy resistance is a major clinical challenge. Herein, we show a molecular classification approach for estimating individual responses to radiotherapy

**Methods:**

Consensus clustering was adopted to classify radiotherapy-sensitive and -resistant clusters in the TCGA-BRCA cohort based upon prognostic differentially expressed radiotherapy response-related genes (DERRGs). The stability of the classification was proven in the GSE58812 cohort via NTP method and the reliability was further verified by quantitative RT-PCR analyses of DERRGs. A Riskscore system was generated through Least absolute shrinkage and selection operator (LASSO) analysis, and verified in the GSE58812 and GSE17705. Treatment response and anticancer immunity were evaluated via multiple well-established computational approaches.

**Results:**

We classified breast cancer patients as radiotherapy-sensitive and -resistant clusters, namely C1 and C2, also verified by quantitative RT-PCR analyses of DERRGs. Two clusters presented heterogeneous clinical traits, with poorer prognosis, older age, more advanced T, and more dead status in the C2. The C1 tumors had higher activity of reactive oxygen species and response to X-ray, proving better radiotherapeutic response. Stronger anticancer immunity was found in the C1 tumors that had rich immune cell infiltration, similar expression profiling to patients who responded to anti-PD-1, and activated immunogenic cell death and ferroptosis. The Riskscore was proposed for improving patient prognosis. High Riskscore samples had lower radiotherapeutic response and stronger DNA damage repair as well as poor anticancer immunity, while low Riskscore samples were more sensitive to docetaxel, doxorubicin, and paclitaxel.

**Conclusion:**

Our findings propose a novel radiotherapy response classification system based upon molecular profiles for estimating radiosensitivity for individual breast cancer patients, and elucidate a methodological advancement for synergy of radiotherapy with ICB.

## Introduction

Breast cancer is a significant global health concern, accounting for a substantial number of cancer-related deaths among women worldwide. The latest global cancer burden data released by the World Health Organization’s International Agency for Research on Cancer (IARC) for 2020 shows that there are 2.26 million new breast cancer cases worldwide (1). About 13% of women are likely to be diagnosed with breast cancer in their lifetime, and the rate of increase is 0.3% per year, which seriously endangers the health of patients (2).

Despite advancements in early detection and treatment strategies, a considerable proportion of breast cancer patients experience disease recurrence and metastasis, leading to poor clinical outcomes. As a fundamental component of breast cancer therapy, 75% of breast cancer patients will receive radiation therapy, which is employed to eradicate subclinical tumor lesions and reduce the risk of local recurrence (3). Research data suggest that radiotherapy can improve the local control of breast cancer by 60% to 70%, and increase the absolute survival rate by 10% (3). However, radiotherapy resistance remains a major clinical challenge, limiting its efficacy and compromising patient outcomes (4).

In recent years, molecular profiling techniques have revolutionized cancer research by providing a comprehensive view of the molecular landscape of tumors (5). According to the status of estrogen receptor (ER), progesterone receptor (PR), ki-67, and HER-2 expression, breast cancer can be classified into four molecular subtypes: Luminal A, Luminal B, HER-2 over-expression, and triple-negative breast cancer. This has become a model for the precision treatment of breast cancer (6). Although this molecular subtyping has been extensively studied in the context of prognosis and treatment selection, there is a paucity of research focusing specifically on molecular classification for estimating radiotherapy response in early breast cancer patients (7).

Insight of the molecular mechanisms underlying radiotherapy response in breast cancer is crucial for improving treatment outcomes and personalizing patient management. Traditionally, clinical factors such as tumor stage, histological grade, and hormone receptor status have been used to guide treatment decisions. However, these factors often fail to accurately predict individual responses to radiotherapy. With the progress of molecular medicine radiotherapy technology, the radiotherapy of breast cancer is also developing towards the direction of individualization. Revealing the radioresistance of breast cancer by molecular mechanism has become a hot topic in radiotherapy research. Radioresistance is a complex process that is generally associated with radiation-induced DNA damage repair, cell cycle dysregulation, cancer stem cell properties, and epithelial-mesenchymal transition (8). Therefore, there is an increasing interest in identifying molecular markers and developing molecular classification systems that can better evaluate radiotherapy response in breast cancer patients. The ability to accurately predict individual responses to radiotherapy would help identify patients who are likely to benefit from this treatment modality and exclude others from potential side effects of radiotherapy.

In recent years, immunotherapy has become a hot research topic for scholars at home and abroad as a new treatment. Clinical trials have shown that PD-1/PD-L1 inhibitors can improve the anti-tumor efficacy in triple-negative breast cancer (9). Radiotherapy may lead to the overexpression of PD-L1 on tumor cells by activating PI3K/AKT, signal transduction, and transcription factor activation. Moreover, PD-L1 stimulates cell migration and promotes the process of epithelial-mesenchymal transition, thereby inducing radioresistance (10). In the basic research of breast cancer, it has been found that PD-1/PD-L1 inhibitors combined with radiotherapy have a stronger anti-tumor effect than single-mode treatment, and the survival time of mice is significantly higher than that of the control group (11, 12).

The objectives of this study are to classify breast cancer patients into radiotherapy-sensitive and -resistant clusters and assess the clinical and molecular characteristics of these clusters. In this study, we employed consensus clustering analysis to identify distinct clusters of patients based on the expression profiles of radiotherapy response-related genes. The stability of the classification was validated in independent patient cohorts. Furthermore, we developed a Riskscore system using LASSO analysis to improve patient prognosis. Additionally, we evaluated treatment response and anticancer immunity using well-established computational approaches. Our findings provide a methodological advancement for the synergy of radiotherapy with immune checkpoint blockade (ICB), potentially enhancing treatment outcomes for breast cancer patients.

## Materials and methods

### Data acquisition and processing

A total of 3693 radiotherapy response-related genes (RRGs) were acquired from previously published literature (13). **Supplementary Table 1** summarizes the RRG list. RNA sequencing data of The Cancer Genome Atlas (TCGA) Breast Cancer (TCGA-BRCA) were gathered from the Genomic Data Commons (https://portal.gdc.cancer.gov/) utilizing the TCGAbiolinks package (14). Following the removal of samples without complete prognostic information, 995 samples were included. The raw read count data were transformed to transcripts per kilobase million, with further log-2 conversion. From the Gene Expression Omnibus (https://www.ncbi.nlm.nih.gov/geo/), two independent breast cancer cohorts: GSE58812 (n=107) (15) and GSE17705 (n=298) (16) were utilized for external verification. The two microarray cohorts were based on the Affymetrix platform, and background correction and standardization were implemented via a robust multiarray averaging approach utilizing the affy package (17). Basic information and demographic data of TCGA-BRCA, GSE58812, and GSE17705 datasets were summarized in **Supplementary Table 2**.

### Selection of differentially expressed RRGs

Differential expression analysis on RRGs was conducted by comparing breast cancer patients who received radiotherapy and those who did not receive radiotherapy. This analysis was achieved via limma package (18). DERRGs were identified with adjusted p-value<0.05.

### Genomic variation analysis

Copy number variation (CNV) data were gathered from the Fire Browse (http://firebrowse.org/). The GISTIC2.0 computational method was adopted for the estimation of gene gains and losses (19). Somatic mutation profiling was obtained from cBioPortal (https://www.cbioportal.org/) and was analyzed based on the matfools package (20).

### Functional enrichment analysis

Gene Ontology (GO) and Kyoto Encyclopedia of Genes and Genomes (KEGG) enrichment analyses were achieved utilizing clusterProfiler (21). The gene sets of reactive oxygen species (ROS), response to X-ray and DNA damage repair pathways were gathered from the Molecular Signatures Database (22). The enrichment score was quantified via single-sample gene set enrichment analysis (ssGSEA) (23).

### Consensus clustering analysis

Univariate-cox regression analysis on DERRGs with prognosis was carried out. DERRGs with p-value<0.05 were employed for consensus clustering analysis via the ConsensusClusterPlus package (24). The optimal number of clusters and stability were determined based on the consensus matrix heatmap, the proportion of ambiguous clustering (PAC), and principal component analysis (PCA). The repeatability and accuracy of the classification were validated via nearest template prediction (NTP) analysis (25). This analysis was conducted based on the top 100 upregulated markers of each cluster in the GSE58812 cohort using the CMScaller package (26).

### Estimation of immune cell infiltration

Eight computational approaches: ssGSEA (23), TIMER (27), CIBERSORT (28), CIBERSORT-ABS (29), QUANTISEQ (30), MCPCOUNTER (31), XCELL (32), and EPIC (33) were utilized for estimation of the abundance of diverse immune cell types.

### Anticancer immunity analysis

Tumor Immune Dysfunction and Exclusion (TIDE) was employed for estimating immune-checkpoint blockade (ICB) response based upon immune evasion mechanisms (34). Response to PD-1 or CTLA4 antibody (35, 36) was inferred via subclass mapping (Submap) approach (37). Immunogenic cell death, ferroptosis, and immune checkpoint molecules were also measured to reveal anticancer immunity.

### Least absolute shrinkage and selection operator analysis

Using the glmnet package (38), LASSO analysis was implemented based on prognostic DERRGs. After identifying the minimum lambda, DERRGs with coefficient ≠0 were further selected through predict.cv.glmnet function. Riskscore was subsequently defined through a combination of expressions of the selected DERRGs with given coefficients. Patients were stratified into low- and high-Riskscore groups with the median Riskscore. The reliability and stability of the model were proven in the GSE58812 and GSE17705 datasets.

### Nomogram establishment

Uni- and multivariate-cox regression analyses on RiskScore, and clinical variables with TCGA-BRCA prognosis were implemented. Based upon independent prognostic variables, a nomogram was generated utilizing the rms package. Additionally, calibration curves were plotted for comparing the nomogram-estimated and actual outcomes.

### Prediction of transcription factors

By adopting the NetworkAnalyst online tool (http://www.networkanalyst.ca) (39), a transcription factor-gene interaction network was visualized.

### Drug sensitivity analysis

Half-maximal inhibitory concentration (IC50) of chemotherapy agents was computed via pRRophetic analysis (40) based upon the Genomics of Drug Sensitivity data (41).

### Drug-gene interaction analysis

Drugs that potentially targeted the DERRGs from the Riskscore were estimated based on the Drug-Gene Interaction Database (www.dgidb.org) (42), and a drug-gene interaction network was generated utilizing the Cytoscape tool (43).

### Reverse transcription and quantitative RT-PCR analyses

Total RNA was isolated from tissues using Trizol (Invitrogen, Carlsbad, CA, USA) following the manufacturer’s protocol. Total RNAs of 0.5 to 1 mg were used as templates for reverse transcription using poly-(T)_20_ primers and M-MLV reverse transcriptase (Promega, Madison, WI, USA). Quantitative RT-PCR (RT-qPCR) was conducted using SYBR Green Mix according to the manufacturer’s protocol (BioRad, Hercules, CA, USA). The primers for cDNA detection are as follows: CIITA, 5’-TGAGGCTGTGTGCTTCTGAG-3’ and 5’-ACACTGTGAGCTGCCTTGG-3’; IL27RA,5’-AGGGAGGAATTAGCACCCCT-3’ and 5’-TGCACACAAGGTGTAGTGGG-3’; ZFP41, 5’-TACCTGGATGGACTTGGGACA-3’ and 5’-GGATGTCCTGCCCTGAATG-3’; N4BP3,5’-GCCTTGCAGGAGGGTTCAAA-3’ and 5’-AGGCAGCTGCTTCATGGTG-3’; GAPDH,5’-GATTCCACCCATGGCAAATTC-3’ and 5’-AGCATCGCCCCACTTGATT-3’.

The breast cancer samples were classified into the resistant and sensitive groups based on their response to radiotherapy, where patients with complete response (CR), partial response (PR), and stable disease (SD) comprised the radiotherapy sensitive group (Rad-S, n=10) and progressive disease (PD) belonged to the radiotherapy resistant group (Rad-R, n=8). The clinical pathological characteristics of the patients are listed in **Supplementary Table 3**.

The tissues were obtained from breast cancer patients participating in a previous clinical study in Tongji Hospital. The ethics approval was not required by the Ethics Committee of Tongji Hospital Affiliated with Tongji Medical College of Huazhong University of Science and Technology, because the clinical study was previously approved by the Ethics Committee.

### Statistical analysis

Data were analyzed via appropriate R packages (version 3.6.1). Continuous variables between two groups were analyzed with Student’s t-test or Wilcoxon test, while categorical data were evaluated via the chi-square test. Pearson’s test was used for correlation analysis. Survival analysis was executed through Kaplan–Meier curves and log-rank test utilizing the survival package. The specificity and sensitivity were appraised via receiver operating characteristic curves (ROCs), and the area under the curve (AUC) was quantified utilizing the pROC package. P-value<0.05 was statistically significant.

## Results

### Multi-omics analysis of DERRGs in breast cancer

**Figure 1:**
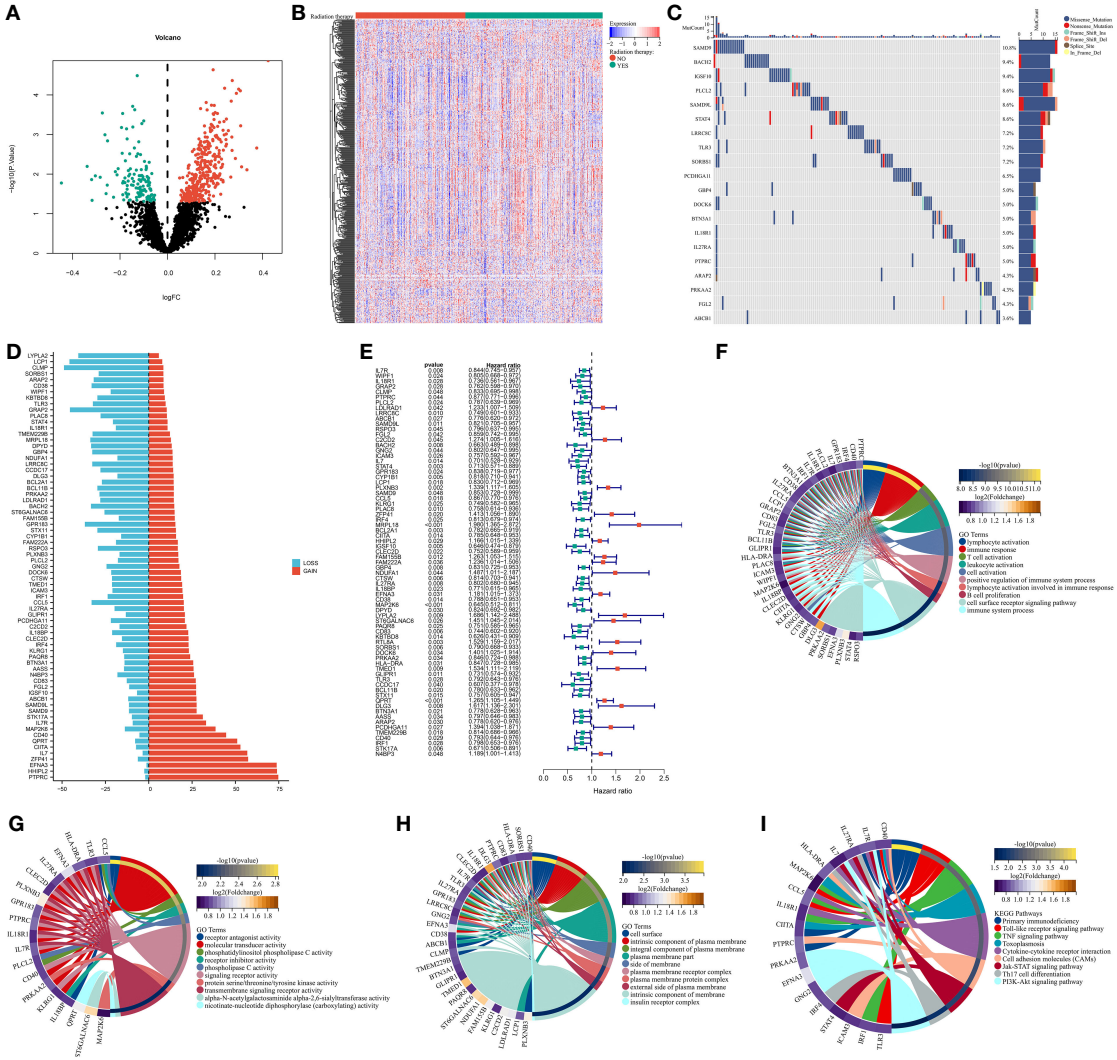
Multi-omics analysis of DERRGs in breast cancer. **(A, B)** Identification of DERRGs through comparing patients with radiotherapy with those without radiotherapy. **(C)** Somatically mutated DERRGs across breast cancer. Mutation forms are marked by unique colors, while DERRGs are ranked by mutated frequency. **(D)** Copy number gains and losses of DERR^©^ **(E)** Univariate-cox regression results on DERRGs with patient survival. **(F–I)** GO and KEGG pathways enriched by DERRGs.

The study firstly determined 125 down-regulated RRGs and up-regulated 341 RRGs in patients with radiotherapy versus those without radiotherapy (p-value<0.05) (**Figures 1A, B**) (**Supplementary Table 4**). These RRGs were considered as DERRGs in breast cancer. Genetic alterations of the DERRGs were subsequently evaluated. Widespread somatic mutations were detected, e.g., SAMD9 (10.8%), BACH2 (9.4%), IGSF10 (9.4%), PLCL2 (8.6%), SAMD9L (8.6%), and STAT4 (8.6%) (**Figure 1C**). Gene gains and losses also frequently occurred such as LCP1, CLMP, GRAP2, PTPRC, HHIPL2, and EFNA3 (**Figure 1D**). The genetic alterations potentially affected the expression of the DERRGs. Prognostic implications of the DERRGs were also observed. Among them, 73 were significantly connected to patient survival (**Figure 1E**). Furthermore, we probed the molecular mechanisms underlying the prognostic DERRGs. Consequently, anticancer immunity and immune response were notably enriched (**Figures 1F–I**), proving their imperative roles.

### Classification of breast cancer patients into radiotherapy-sensitive and -resistant consensus clusters

**Figure 2:**
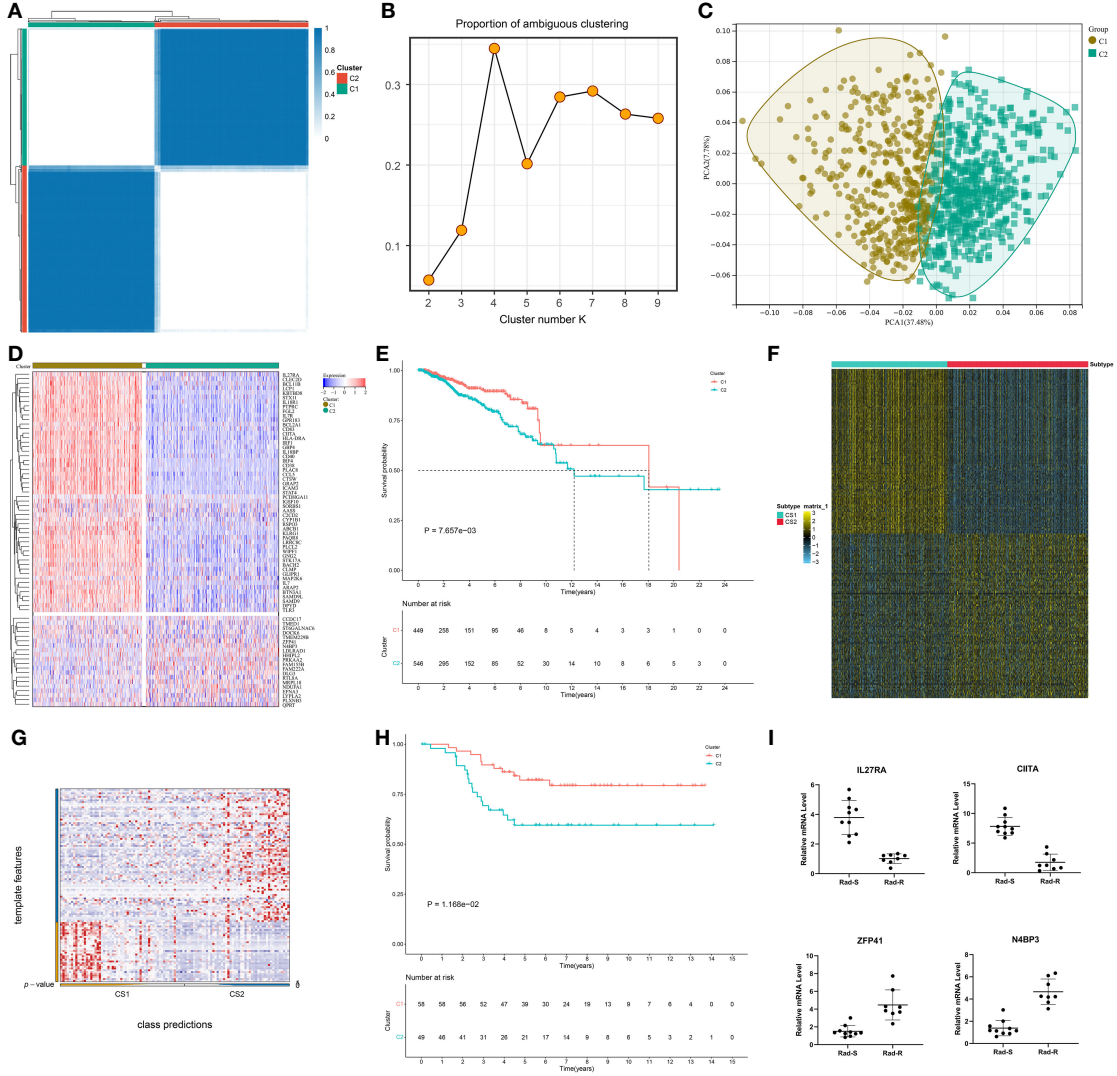
Classification of breast cancer patients into radiotherapy-sensitive and -resistant consensus clusters. **(A)** Consensus matrix heatmap at cluster number k=2. White means samples never get together; blue means the samples are always clustered together. **(B)** PAC distribution at cluster numbers k=2~9. **(C)** PCA verifying the different transcriptome profiles in two clusters. **(D)** Expression values of the prognostic DERRGs in two clusters. Blue to red denotes down- to up-regulated expre^©^on. **(E)** Survival probability of two clusters. **(F)** The top 100 up-regulated marker genes in each cluster. **(G, H)** Verification of the patient classification, and survival difference via NTP method in the GSE58812 cohort. **(I)** Comparison of CIITA, 27RA, ZFP41, and N4BP3 expression in breast cancer tissues that are sensitive (n=10) or resistant (n=8) to radiotherapy by quantitative real-time PCR detection.

TCGA-BRCA samples were classified as two consensus clusters based on the transcriptome values of the prognostic DERRGs (**Figure 2A**). PAC score was relatively small at cluster number k=2 (**Figure 2B**). This demonstrated that the optimal k value was 2. PCA also unveiled the diverse transcriptome profiling in two clusters (**Figure 2C**). Many radiotherapy-sensitive genes were up-regulated in the C1 tumors, while radiotherapy-resistant genes were up-regulated in another cluster (**Figure 2D**). Thus, we inferred the C1 tumors were regarded as radiotherapy-sensitive clusters, while the C2 tumors were regarded as radiotherapy-resistant clusters. Worse survival was detected in the C2 versus C1 patients (**Figure 2E**). The GSE58812 dataset was adopted to prove the classification. The top 100 up-regulated marker genes in each consensus cluster were selected (**Figure 2F**; **Supplementary Table 5**), and samples with p-value<0.05 were extracted for quantification and assessment of prediction confidence (**Figure 2G**). Consistently, the C2 posessed a less favorable prognosis than the C1 (**Figure 2H**). In addition, the mRNA levels of CIITA and IL27RA are significantly downregulated, while the mRNA levels of ZFP41 and N4BP3 are significantly increased in radiotherapy-sensitive tumors, rather than radiotherapy-resistant tumors (**Figure 2I**). The detection results are consistent with the predictions of this system. Hence, the classification was reliable and repeatable.

### Two consensus clusters are characterized by different clinical traits and radiotherapy responses

**Figure 3:**
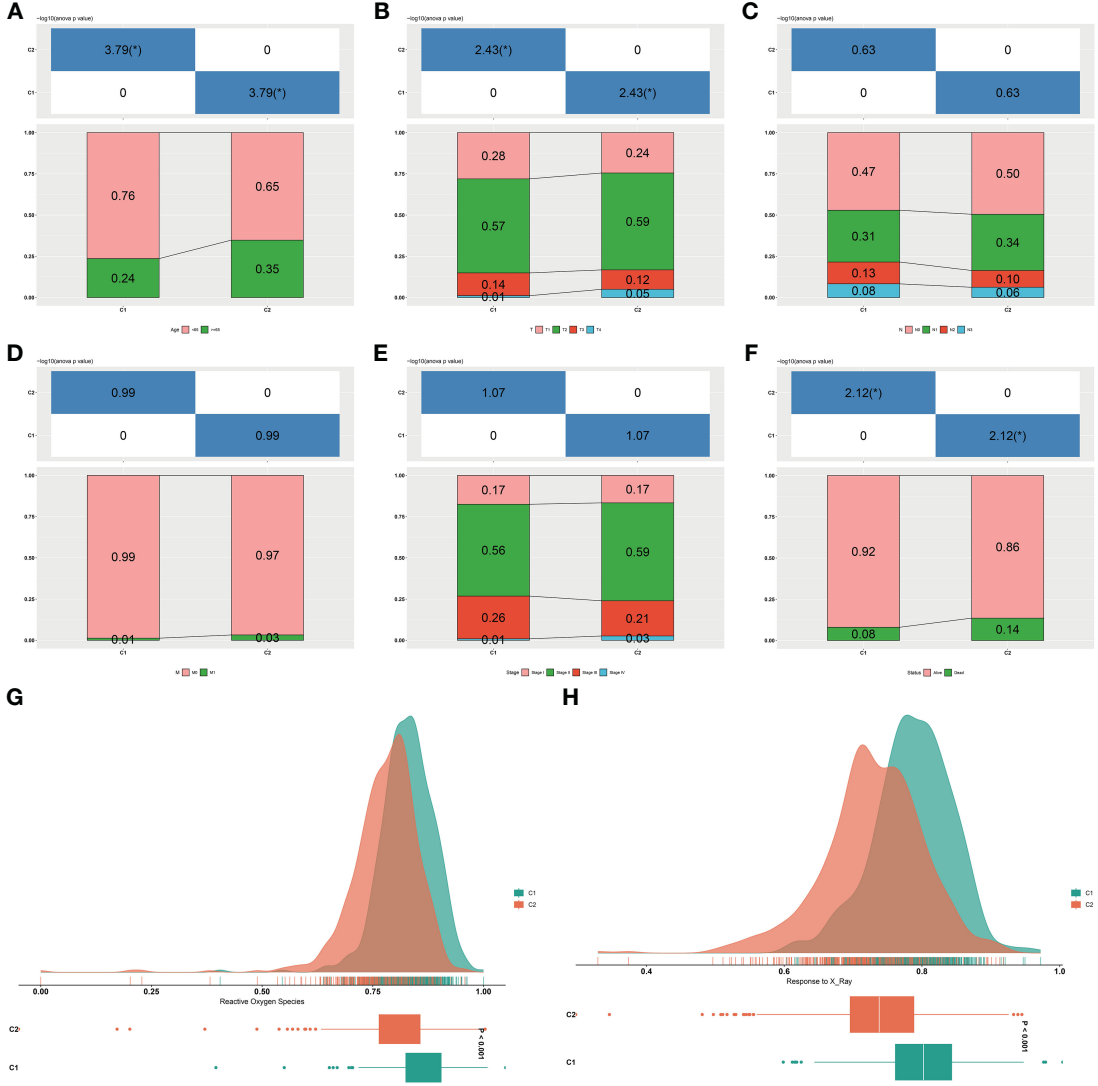
Two consensus clusters are characterized by different clinical traits and radiotherapy responses. **(A–F)** Diverse age, T, N, M, stage, and survival status features in two clusters. *P-value<0.05. **(G, H)** Difference ROS and response to X-ray between two clusters.

The C2 had more cases with age ≥65, more advanced T, and dead status in comparison to the C1, but without difference in N, M, and stage (**Figures 3A–F**). Radiotherapy results in DNA damage directly through ionization or indirectly through generating ROS, thus destroying tumor cells (44). The C1 tumors posessed stronger ROS activity and responses to X-ray (**Figures 3G, H**), further demonstrating that patients in this cluster better responded to radiotherapy.

### Two consensus clusters show diverse genomic mutation features

**Figure 4:**
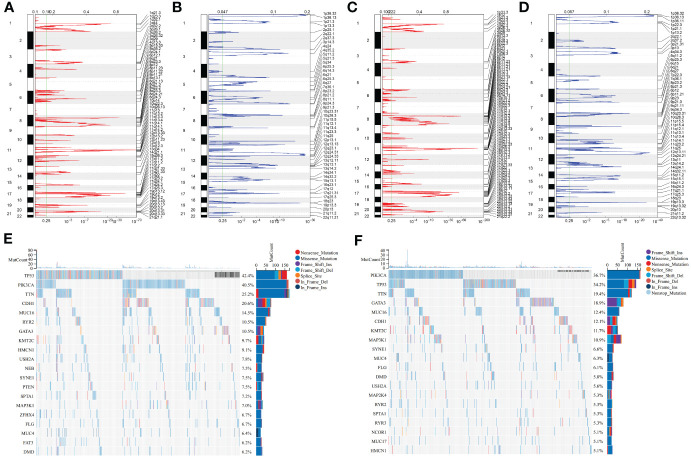
Two consensus clusters show diverse genomic alterations. **(A, B)** Gene gains and losses in the C1 tumors. The significance was set as q-value<0.25. **(C, D)** Gene gains and losses in the C2 tumors. **(E, F)** Dominating somatically mutated genes in two clusters. Mutation forms are represented by unique colors. Genes are ranked by mutation frequency.

Genomic mutation profiles in two clusters were investigated. In the C1 tumors, 2102 and 4776 genes experienced copy number gains and losses, respectively (**Figures 4A, B**). Meanwhile, in the C2 tumors, 2656 and 5552 genes had copy number gains and losses, respectively (**Figures 4C, D**). Overall, CNVs were more frequent in the C2. Somatic mutations were found to be more frequent in the C1 versus C2 tumors, e.g., TP53: 42.4% versus 34.2%, PIK3CA: 40.5% versus 36.7%, TTN: 25.2% versus 19.4% (**Figures 4E, F**).

### Two consensus clusters present heterogeneous anticancer immunity and immune escape

**Figure 5:**
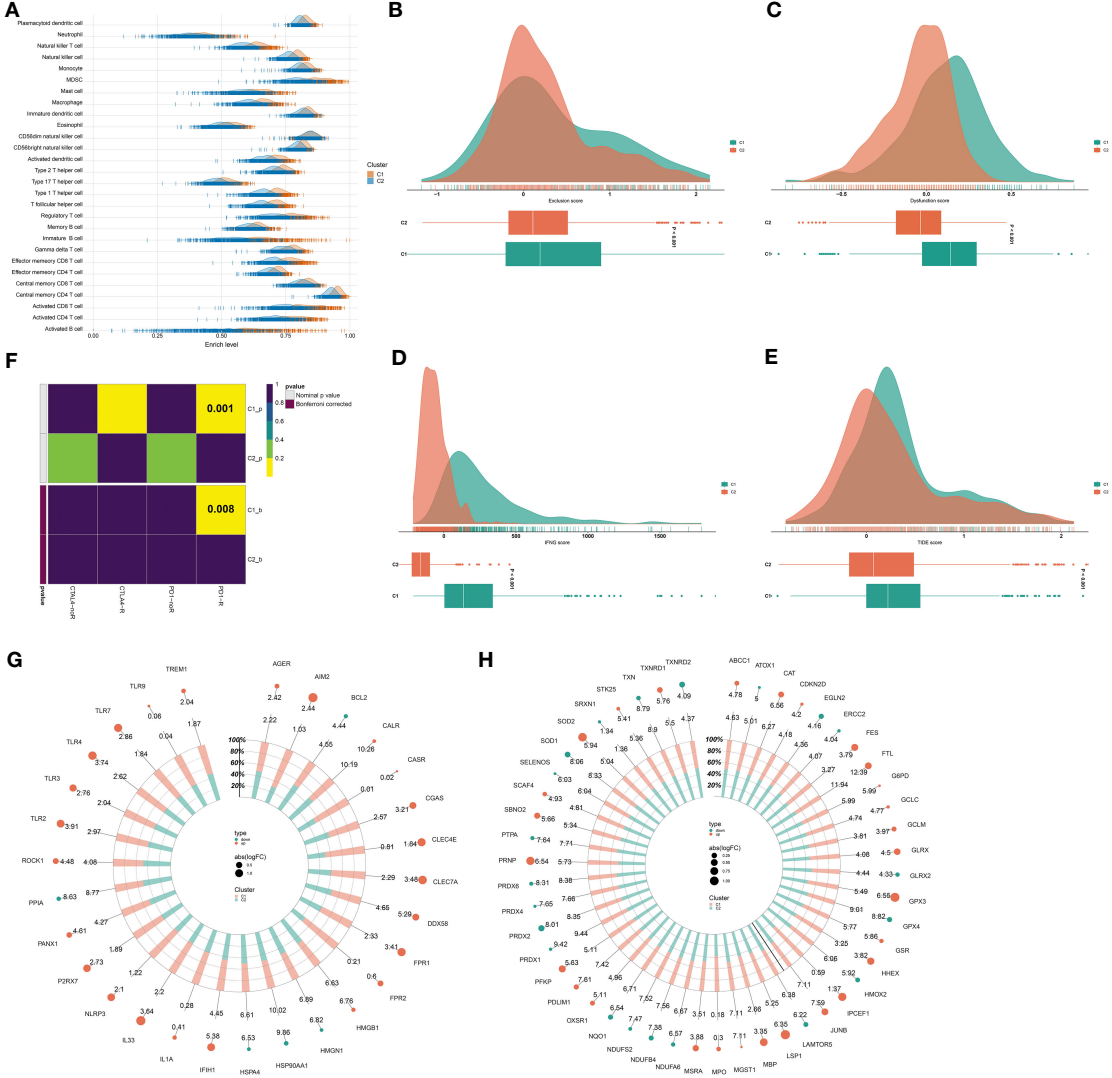
Two consensus clusters exhibit heterogeneous anticancer immunity and immune evasion mechanisms. **(A)** Abundance of immune cell types in two clusters. **(B–E)** Different exclusion, dysfunction, IFNG, and TIDE scores in two clusters. **(F)** Submap for investigating the similarity in the expression profiling of two clusters with that of samples that responded or did not respond to PD-1 or CTLA4 antibody. **(G, H)** Difference in immunogenic cell death and ferroptosis molecules between two clusters.

Most immune cell populations (e.g., natural killer cells, natural killer T cells, activated dendritic cells, activated CD4 and CD8 T cells) displayed more infiltration in the C1 versus another cluster, demonstrating stronger anticancer immunity in C1 tumors (**Figure 5A**). Lower exclusion, dysfunction, IFNG, and TIDE scores were found in C2 tumors, inferring patients in the cluster potentially responded to ICB (**Figures 5B–E**). In addition, the C1 exhibited a similar expression profile to that of samples responding to the PD-1 antibody (**Figure 5F**), further proving stronger anticancer immunity in C1 tumors. Immunogenic cell death that can be induced by radiotherapy exerts a crucial role in evoking systemic immune response against tumors (45). Many immunogenic cell death molecules, e.g., TLR2/3/4/7/9, CALR, CGAS, CLEC4E, CLEC7A, DDX58, FPR1/2, HMGB1, IL33, NLPR3, and P2RX7 were notably up-regulated the C1 (**Figure 5G**), revealing activated immunogenic cell death in the cluster. Ferroptosis has been implicated in anticancer immunity and immune response (46). The C1 presented remarkable up-regulation of many ferroptosis molecules, such as LSP1, CAT, SOD2, PRNP, FTL, FES, GLRX, PFKP, PDIM1, MBP, HHEX, and GPX3 (**Figure 5H**). Overall, the C1 tumors were characterized by potent anticancer immunity.

### Generation of a radiotherapy response-based RiskScore model for improving prognosis

**Figure 6:**
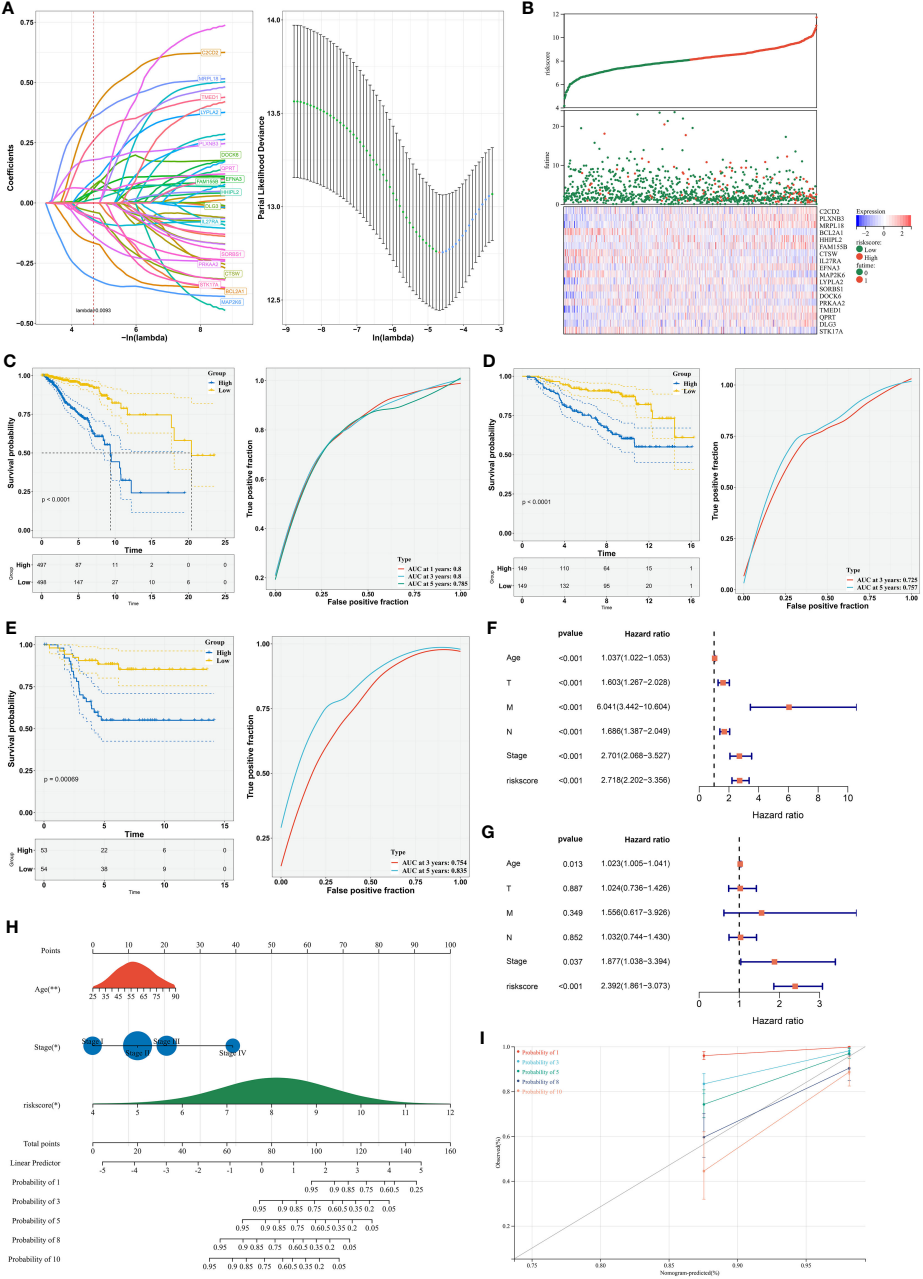
Establishment of a radiotherapy response-based RiskScore model for improving prognosis. **(A)** LASSO coefficients of the DERRGs and selection of the minimum lambda value. **(B)** Distribution of RiskScore, survival duration, and expression of the DERRGs. **(C)** Survival probability of low- and high-RiskScore groups and 1-, 3-, and 5-year ROCs. **(D, E)** Verification of survival outcomes and ROCs in the GSE17705 and GSE58812 cohorts. **(F, G)** Uni- and multivariate-cox regression analyses on the RiskScore and clinical parameters with prognosis. **(H)** Nomogram generation based upon independent prognostic variables. *, P-value<0.05; **, P-value<0.01. **(I)** Calibration curves for verifying the prediction accuracy of the nomogram.

LASSO analysis on the prognostic DERRGs was conducted. In line with the minimum lambda (0.0093), a radiotherapy resistance-based RiskScore model was generated following the RiskScore = 0.614 * C2CD2 + 0.197 * PLXNB3 + 0.443 * MRPL18 + (-0.212) * BCL2A1 + 0.037 * HHIPL2 + 0.093 * FAM155B + (-0.07) * CTSW + (-0.144) * IL27RA + 0.13 * EFNA3 + (-0.381) * MAP2K6 + 0.203 * LYPLA2 + (-0.143) * SORBS1 + 0.228 * DOCK6 + (-0.248) * PRKAA2 + 0.431 * TMED1 + 0.044 * QPRT + 0.199 * DLG3 + (-0.097) * STK17A (**Figure 6A**). Using the formula, the RiskScore of each TCGA-BRCA sample was computed. Under the median RiskScore, TCGA-BRCA samples were stratified into two groups (**Figure 6B**). Survival difference was found in two groups, with poorer survival probability for the high RiskScore group versus another group (**Figure 6C**). ROCs were plotted to appraise the prediction efficiency. The one-, three-, and five-year AUC values were 0.785, 0.800, and 0.800, respectively, proving that the RiskScore was capable of estimating patient prognosis. The excellent efficacy was externally demonstrated in the GSE17705 and GSE58812 datasets (**Figures 6D, E**).

Uni- in combination with multivariate-cox regression results demonstrated the independency of the RiskScore in prognostication in addition to age and stage (**Figures 6F, G**). Based upon them, a nomogram was generated for estimation of one-, three-, five-, eight-, and ten-year survival probability (**Figure 6H**). To verify its performance, calibration curves were conducted. Consequently, the nomogram-estimated prognosis was close to the actual outcomes (**Figure 6I**). Overall, our nomogram provided a method to estimate survival outcomes via integration of the RiskScore, age, and stage.

### Transcriptional regulatory mechanisms underlying the RiskScore

**Figure 7:**
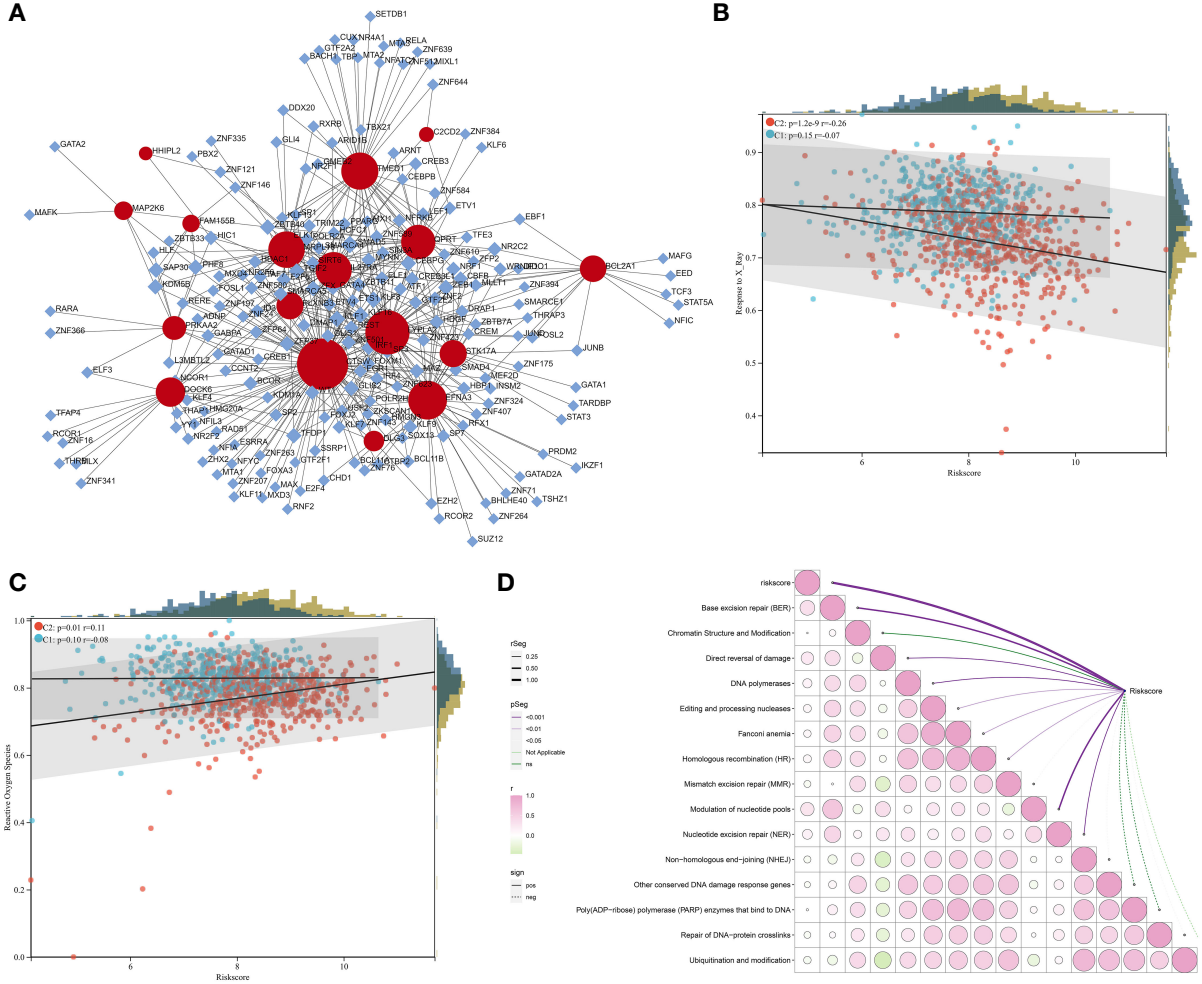
Transcriptional regulatory mechanisms underlying the RiskScore and a positive association of the RiskScore with radiotherapy resistance. **(A)** Transcriptional factor-gene network. Rhombus, transcriptional factor; circle, the DERRG from the RiskScore. **(B, C)** Relationships of the RiskScore with response to X-ray and ROS in the C1 and C2 tumors. **(D)** Correlation between the RiskScore with DNA damage repair pathways. The bubble from green to pink denotes the association from negative to positive. P-value is represented by a unique line color.

Transcriptional factors of the DERRGs from the RiskScore were then investigated. As illustrated in **Figure 7A**, DOCK6, FAM155B, MAP2K6, HHIPL2, PRKAA2, MRPL18, PLXNB3, IL27RA, CTSW, DLG3, LYPLA2, TMED1, QPRT, EFNA3, STK17A, C2CD2, and BCL2A1 were transcriptionally modulated by multiple transcriptional factors.

### The RiskScore positively correlates to radiotherapy resistance

The RiskScore was negatively connected to response to X-ray, especially for the C2 tumors (**Figure 7B**). A positive relationship between the RiskScore and ROS was also found in the C2 tumors (**Figure 7C**). In addition, the RiskScore was positively linked with DNA damage response pathways, e.g., base excision repair, direct reversal of damage, homologous recombination, and nucleotide excision repair (**Figure 7D**). Altogether, the RiskScore was positively connected to radiotherapy resistance.

### The RiskScore is negatively associated with anticancer immunity

**Figure 8:**
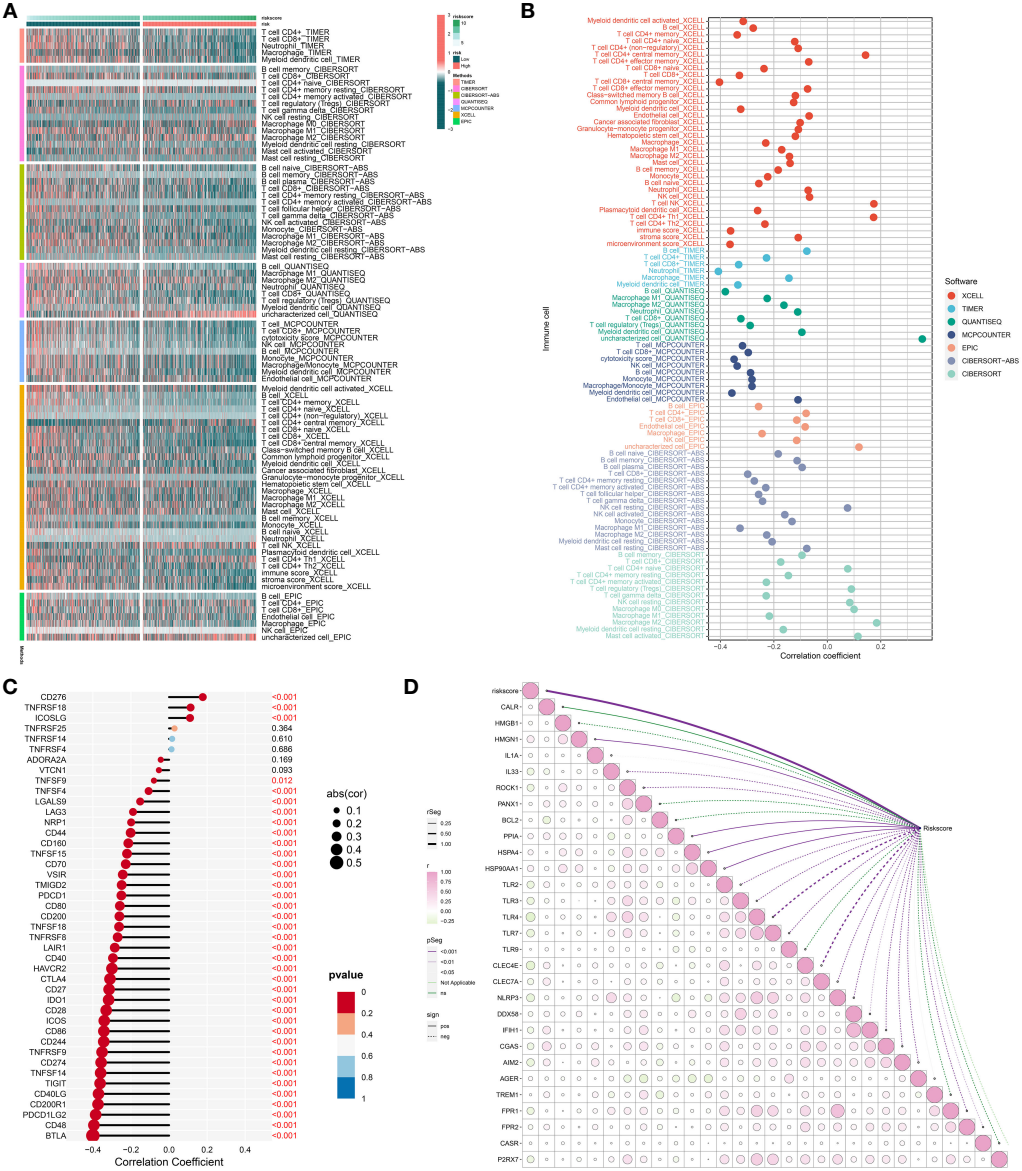
Association of the RiskScore with immunogenicity. **(A)** Distribution of the infiltration of distinct immune cell populations in low- and high-RiskScore groups by use of multiple algorithms. Green to red denotes weak to strong infiltration. **(B)** Association of the RiskScore with infiltrative immune cells. The bubble location indicates the correlation coefficient. **(C)** Correlation between the RiskScore with known immune checkpoints. The bigger the bubble, the stronger the correlation. **(D)** Relationships of the RiskScore with immunogenic cell death molecules. The bubble from green to pink shows the correlation from negative to positive. P-value is represented by a unique line color.

The study executed multiple well-established computational methods to estimate the abundance of immune cells. Intriguingly, most immune cells, notably cytotoxic T-lymphocytes that are critical effectors of anticancer immunity (47), presented higher infiltration in low-RiskScore tumors (**Figure 8A**). Moreover, the RiskScore was negatively linked with cytotoxic T-lymphocytes (**Figure 8B**), indicative of a negative association of the RiskScore with anticancer immunity. We also focused on the remarkably negative relationships of the RiskScore with common immune checkpoint molecules, e.g., PDCD1 (PD-1) and its ligands CD274 (PD-L1) and PDCD1LG2 (PD-L2) (**Figure 8C**) (48). This indicated that low-RiskScore tumors displayed a stronger sensitivity to ICB. The RiskScore was also found to be negatively connected to most immunogenic cell death molecules, such as IL33, TLR2/3/4/7, CLEC4E, NLRP3, CGAS, and FPR1/2 (**Figure 8D**), demonstrating a negative association of the RiskScore with immunogenic cell death.

### Low-RiskScore patients are more sensitive to chemotherapy and several DERRGs in the RiskScore model are potential druggable targets

**Figure 9:**
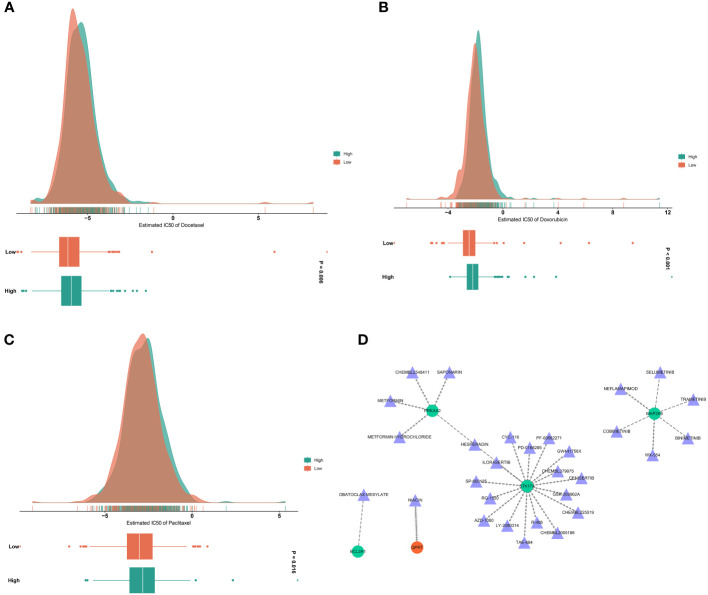
Analysis of drug sensitivity and druggable targets. **(A–C)** Estimated IC50 values of docetaxel, doxorubicin, and paclitaxel in low- and high-RiskScore groups. The larger the IC50, the weaker the sensitivity to a drug. **(D)** Drug-gene network. Triangle, drug; circle, the DERRG from the RiskScore model.

Docetaxel, doxorubicin, and paclitaxel are routinely applied in chemotherapy for breast cancer. Here, we evaluated the difference in sensitivity to these chemotherapeutic agents. Consequently, the low RiskScore group presented significantly lower IC50 values of docetaxel, doxorubicin, and paclitaxel versus the high RiskScore group (**Figures 9A–C**). Hence, low-RiskScore patients were more sensitive to the above chemotherapeutic drugs.

Small molecules that potentially targeted the DERRGs from the RiskScore model were also analyzed. As illustrated in **Figure 9D**, seventeen small molecules: HESPERADIN, SP-600125, CHEMBL225519, LY-2090314, R-406, GW441756X, CYC-116, CHEMBL379975, PD-0166285, CHEMBL2005186, ILORASERTIB, GSK-269962A, AZD-1080, PF-00562271, TAE-684, RG-1530, and CENISERTIB specifically targeted STK17A; six small molecules: TRAMETINIB, SELUMETINIB, COBIMETINIB, BINIMETINIB, NEFLAMAPIMOD, and WX-554 specifically targeted MAP2K6; five small molecules: HESPERADIN, METFORMIN HYDROCHLORIDE, CHEMBL2348411, METFORMIN, and SAPONARIN specifically targeted PRKAA2; QPRT was a specific target of NIACIN; and BCL2A1 was a specific target of OBATOCLAX MESYLATE. Thus, PRKAA2, STK17A, MAP2K6, BCL2A1, and QPRT were potential druggable targets.

## Discussion

The present study has provided a comprehensive molecular classification system for predicting radiotherapy response and anticancer immunity in individual breast cancer patients. This classification system, based on the consensus clustering of differentially expressed radiotherapy response-related genes (DERRGs), has identified two distinct clusters, C1 and C2, with C1 tumors demonstrating a higher sensitivity to radiotherapy and stronger anticancer immunity. The identification of these two distinct clusters has significant implications for the personalized treatment of breast cancer patients, as it helps the prediction of individual responses to radiotherapy, thereby enabling the decision of treatment strategies to enhance therapeutic efficacy and minimize adverse effects.

The C1 cluster, characterized by a higher activity of Reactive Oxygen Species (ROS) and a stronger response to X-ray, demonstrated a better radiotherapeutic response. ROS accumulation can lead to DNA damage and genomic instability, induce multiple forms of cell death, and play an important role in cancer development and progression (49). In preclinical studies of breast cancer, ROS-induced nanophototherapy systems have been shown to increase radiosensitivity (50). This finding is consistent with previous studies (51) that have highlighted the role of reactive oxygen species in mediating the cytotoxic effects of radiotherapy. Furthermore, the C1 tumors exhibited a stronger anticancer immunity, as evidenced by the rich immune cell infiltration and the similar expression profiling to patients who responded to anti-PD-1. In this study, we validated the high expression of MHC class II transactivator (CIITA) in the C1 population through quantitative PCR. CIITA is a master controller of antigen presentation and a crucial factor in adaptive immunity. In recent years, CIITA has been speculated to be an important exploration target in anti-checkpoint blockade immunotherapy and anti-tumor vaccination (52, 53). Therefore, the C1 tumors may be more sensitive to ICB, which has emerged as a promising therapeutic strategy for various types of cancer. However, the effects of RGGs on chemotherapy response in the C1 population are somewhat contradictory, with some genes enhancing chemotherapy sensitivity (IL7R) (54) while others promoting chemotherapy drug resistance (PLAC8, CCL5) (55, 56).

In contrast, the C2 cluster was associated with poorer prognosis, older age, more advanced T stage, and more dead status. These tumors demonstrated a lower response to radiotherapy and a stronger DNA damage repair capacity, suggesting a higher resistance to radiotherapy. Reactive oxygen species (ROS) are one of the major inducers of DNA damage, and one of the key determinants of the efficacy of anti-tumor therapy is the severity of DNA damage resulting in tumor cells. However, cancer cells are able to adapt DNA repair pathways to make tumors resistant to treatment (57). Inhibitors targeting the DNA repair system may enhance the radiosensitivity of tumors, but a few studies have suggested that the loss of DNA repair function may increase the rate of gene mutation and eventually lead to radioresistance. Therefore, the relationship between DNA damage repair and radiotherapy resistance is still controversial and needs further study (58). This finding underscores the need for alternative therapeutic strategies for patients with C2 tumors, such as the use of DNA damage repair inhibitors to sensitize these tumors to radiotherapy. Moreover, the C2 tumors exhibited poor anticancer immunity, which highlights the need for further research to elucidate the mechanisms underlying the immune escape.

The RiskScore system proposed in this study represents a significant advancement in the prognostication of breast cancer patients. This system, based on the expression of DERRGs, was capable of predicting patient prognosis with high accuracy, as evidenced by the high area under the curve (AUC) values. Importantly, the RiskScore system was independent of age and stage, suggesting that it could provide additional prognostic information beyond these traditional prognostic factors. Furthermore, the RiskScore system was associated with radiotherapy response and anticancer immunity. The high-RiskScore samples demonstrate lower radiotherapeutic response and poor anticancer immunity, while low-RiskScore samples are more sensitive to chemotherapy drugs such as docetaxel, doxorubicin, and paclitaxel. This suggests the RiskScore system could potentially help the decision of therapeutic strategies for individual patients.

In conclusion, this study has proposed a novel radiotherapy response classification system based on molecular profiles for evaluating radiosensitivity in individual breast cancer patients. This system, combined with the RiskScore system, could potentially revolutionize the personalized treatment of breast cancer by enabling the prediction of individual responses to radiotherapy and immunotherapy. However, further validation in prospective clinical trials is warranted to confirm the clinical significance of these systems. Moreover, future research should focus on elucidating the molecular mechanisms underlying the differential radiosensitivity and anticancer immunity of the identified clusters, which could guide the development of new therapeutic targets.

## Data availability statement

The original contributions presented in the study are included in the article/**Supplementary Material**. Further inquiries can be directed to the corresponding authors.

## Ethics statement

The studies involving humans were approved by Tongji Hospital, Tongji Medical College, Huazhong University of Science and Technology, Wuhan, China. The studies were conducted in accordance with the local legislation and institutional requirements. Written informed consent for participation in this study was provided by the participants’ legal guardians/next of kin.

## Author contributions

JZ: Conceptualization, Data curation, Formal Analysis, Funding acquisition, Methodology, Software, Supervision, Validation, Visualization, Writing – original draft. LL: Formal Analysis, Methodology, Software, Validation, Visualization, Writing – review & editing. HS: Data curation, Formal Analysis, Methodology, Software, Validation, Visualization, Writing – review & editing. ZF: Conceptualization, Data curation, Methodology, Project administration, Software, Visualization, Writing – review & editing. TC: Conceptualization, Data curation, Formal Analysis, Funding acquisition, Investigation, Methodology, Project administration, Supervision, Writing – review & editing.

## Supplementary material

Supplementary Table 1 The list of RRGs.

Supplementary Table 2 Patient information in TCGA-BRCA, GSE58812, and GSE17705 cohorts.

Supplementary Table 3 The clinical pathological characteristics of breast cancer patients.

Supplementary Table 4 The list of DERRGs in breast cancer.

Supplementary Table 5 The top 100 up-regulated marker genes in each cluster.

## References

[1] Sung H Ferlay J Siegel RL Laversanne M Soerjomataram I Jemal A. Global cancer statistics 2020: globocan estimates of incidence and mortality worldwide for 36 cancers in 185 countries. CA Cancer J Clin (2021) 71(3):209–49. doi: 10.3322/caac.21660 33538338

[2] DeSantis CE Ma J Gaudet MM Newman LA Miller KD Goding Sauer A. Breast cancer statistics, 2019. CA Cancer J Clin (2019) 69(6):438–51. doi: 10.3322/caac.21583 31577379

[3] Castaneda SA Strasser J. Updates in the treatment of breast cancer with radiotherapy. Surg Oncol Clin N Am (2017) 26(3):371–82. doi: 10.1016/j.soc.2017.01.013 28576177

[4] Wu Y Song Y Wang R Wang T. Molecular mechanisms of tumor resistance to radiotherapy. Mol Cancer (2023) 22(1):96. doi: 10.1186/s12943-023-01801-2 37322433 PMC10268375

[5] Goodwin S McPherson JD McCombie WR. Coming of age: ten years of next-generation sequencing technologies. Nat Rev Genet (2016) 17(6):333–51. doi: 10.1038/nrg.2016.49 PMC10373632 27184599

[6] Harbeck N Penault-Llorca F Cortes J Gnant M Houssami N Poortmans P. Breast cancer. Nat Rev Dis Primers (2019) 5(1):66. doi: 10.1038/s41572-019-0111-2 31548545

[7] Perou CM Sorlie T Eisen MB van de Rijn M Jeffrey SS Rees CA. Molecular portraits of human breast tumours. Nature (2000) 406(6797):747–52. doi: 10.1038/35021093 10963602

[8] Chen GZ Zhu HC Dai WS Zeng XN Luo JH Sun XC. The mechanisms of radioresistance in esophageal squamous cell carcinoma and current strategies in radiosensitivity. J Thorac Dis (2017) 9(3):849–59. doi: 10.21037/jtd.2017.03.23 PMC5394057 28449496

[9] Rizzo A Cusmai A Acquafredda S Giovannelli F Rinaldi L Misino A. Keynote-522, impassion031 and geparnuevo: changing the paradigm of neoadjuvant immune checkpoint inhibitors in early triple-negative breast cancer. Future Oncol (2022) 18(18):2301–9. doi: 10.2217/fon-2021-1647 35378995

[10] Gong X Li X Jiang T Xie H Zhu Z Zhou F. Combined radiotherapy and anti-pd-L1 antibody synergistically enhances antitumor effect in non-small cell lung cancer. J Thorac Oncol (2017) 12(7):1085–97. doi: 10.1016/j.jtho.2017.04.014 28478231

[11] Laack NN Pafundi D Anderson SK Kaufmann T Lowe V Hunt C. Initial results of a phase 2 trial of (18)F-dopa pet-guided dose-escalated radiation therapy for glioblastoma. Int J Radiat Oncol Biol Phys (2021) 110(5):1383–95. doi: 10.1016/j.ijrobp.2021.03.032 33771703

[12] Kang J Pilones KA Daviaud C Kraynak J Formenti SC. Vista blockade immunotherapy in a multi-modal approach to triple negative breast cancer (Tnbc) in mice and impact on microbiome. Int J Radiat OncologyBiologyPhysics (2019) 105(1):S88–S9. doi: 10.1016/j.ijrobp.2019.06.561

[13] Peng SL Wang R Zhou YL Wei W Zhong GH Huang XT. Insight of a metabolic prognostic model to identify tumor environment and drug vulnerability for lung adenocarcinoma. Front Immunol (2022) 13:872910. doi: 10.3389/fimmu.2022.872910 35812404 PMC9262104

[14] Colaprico A Silva TC Olsen C Garofano L Cava C Garolini D. Tcgabiolinks: an R/bioconductor package for integrative analysis of Tcga data. Nucleic Acids Res (2016) 44(8):e71. doi: 10.1093/nar/gkv1507 26704973 PMC4856967

[15] Jézéquel P Loussouarn D Guérin-Charbonnel C Campion L Vanier A Gouraud W. Gene-expression molecular subtyping of triple-negative breast cancer tumours: importance of immune response. Breast Cancer Res (2015) 17:43. doi: 10.1186/s13058-015-0550-y 25887482 PMC4389408

[16] Symmans WF Hatzis C Sotiriou C Andre F Peintinger F Regitnig P. Genomic index of sensitivity to endocrine therapy for breast cancer. J Clin Oncol (2010) 28(27):4111–9. doi: 10.1200/jco.2010.28.4273 PMC2953969 20697068

[17] Gautier L Cope L Bolstad BM Irizarry RA. Affy–analysis of affymetrix genechip data at the probe level. Bioinformatics (2004) 20(3):307–15. doi: 10.1093/bioinformatics/btg405 14960456

[18] Ritchie ME Phipson B Wu D Hu Y Law CW Shi W. Limma powers differential expression analyses for Rna-sequencing and microarray studies. Nucleic Acids Res (2015) 43(7):e47. doi: 10.1093/nar/gkv007 25605792 PMC4402510

[19] Mermel CH Schumacher SE Hill B Meyerson ML Beroukhim R Getz G. Gistic2.0 facilitates sensitive and confident localization of the targets of focal somatic copy-number alteration in human cancers. Genome Biol (2011) 12(4):R41. doi: 10.1186/gb-2011-12-4-r41 21527027 PMC3218867

[20] Mayakonda A Lin DC Assenov Y Plass C Koeffler HP. Maftools: efficient and comprehensive analysis of somatic variants in cancer. Genome Res (2018) 28(11):1747–56. doi: 10.1101/gr.239244.118 PMC6211645 30341162

[21] Yu G Wang LG Han Y He QY. Clusterprofiler: an R package for comparing biological themes among gene clusters. Omics (2012) 16(5):284–7. doi: 10.1089/omi.2011.0118 PMC3339379 22455463

[22] Liberzon A Birger C Thorvaldsdóttir H Ghandi M Mesirov JP Tamayo P. The molecular signatures database (Msigdb) hallmark gene set collection. Cell Syst (2015) 1(6):417–25. doi: 10.1016/j.cels.2015.12.004 PMC4707969 26771021

[23] Hänzelmann S Castelo R Guinney J. Gsva: gene set variation analysis for microarray and Rna-seq data. BMC Bioinf (2013) 14:7. doi: 10.1186/1471-2105-14-7 PMC3618321 23323831

[24] Wilkerson MD Hayes DN. Consensusclusterplus: A class discovery tool with confidence assessments and item tracking. Bioinformatics (2010) 26(12):1572–3. doi: 10.1093/bioinformatics/btq170 PMC2881355 20427518

[25] Hoshida Y. Nearest template prediction: A single-sample-based flexible class prediction with confidence assessment. PLoS One (2010) 5(11):e15543. doi: 10.1371/journal.pone.0015543 21124904 PMC2990751

[26] Eide PW Bruun J Lothe RA Sveen A. Cmscaller: an R package for consensus molecular subtyping of colorectal cancer pre-clinical models. Sci Rep (2017) 7(1):16618. doi: 10.1038/s41598-017-16747-x 29192179 PMC5709354

[27] Li T Fan J Wang B Traugh N Chen Q Liu JS. Timer: A web server for comprehensive analysis of tumor-infiltrating immune cells. Cancer Res (2017) 77(21):e108–e10. doi: 10.1158/0008-5472.Can-17-0307 PMC6042652 29092952

[28] Newman AM Liu CL Green MR Gentles AJ Feng W Xu Y. Robust enumeration of cell subsets from tissue expression profiles. Nat Methods (2015) 12(5):453–7. doi: 10.1038/nmeth.3337 PMC4739640 25822800

[29] Tian Y Xiao H Yang Y Zhang P Yuan J Zhang W. Crosstalk between 5-methylcytosine and N(6)-methyladenosine machinery defines disease progression, therapeutic response and pharmacogenomic landscape in hepatocellular carcinoma. Mol Cancer (2023) 22(1):5. doi: 10.1186/s12943-022-01706-6 36627693 PMC9830866

[30] Finotello F Mayer C Plattner C Laschober G Rieder D Hackl H. Molecular and pharmacological modulators of the tumor immune contexture revealed by deconvolution of Rna-seq data. Genome Med (2019) 11(1):34. doi: 10.1186/s13073-019-0638-6 31126321 PMC6534875

[31] Becht E Giraldo NA Lacroix L Buttard B Elarouci N Petitprez F. Estimating the population abundance of tissue-infiltrating immune and stromal cell populations using gene expression. Genome Biol (2016) 17(1):218. doi: 10.1186/s13059-016-1070-5 27765066 PMC5073889

[32] Aran D Hu Z Butte AJ. Xcell: digitally portraying the tissue cellular heterogeneity landscape. Genome Biol (2017) 18(1):220. doi: 10.1186/s13059-017-1349-1 29141660 PMC5688663

[33] Racle J Gfeller D. Epic: A tool to estimate the proportions of different cell types from bulk gene expression data. Methods Mol Biol (2020) 2120:233–48. doi: 10.1007/978-1-0716-0327-7_17 32124324

[34] Jiang P Gu S Pan D Fu J Sahu A Hu X. Signatures of T cell dysfunction and exclusion predict cancer immunotherapy response. Nat Med (2018) 24(10):1550–8. doi: 10.1038/s41591-018-0136-1 PMC6487502 30127393

[35] Balar AV Galsky MD Rosenberg JE Powles T Petrylak DP Bellmunt J. Atezolizumab as first-line treatment in cisplatin-ineligible patients with locally advanced and metastatic urothelial carcinoma: A single-arm, multicentre, phase 2 trial. Lancet (2017) 389(10064):67–76. doi: 10.1016/s0140-6736(16)32455-2 27939400 PMC5568632

[36] Chen PL Roh W Reuben A Cooper ZA Spencer CN Prieto PA. Analysis of immune signatures in longitudinal tumor samples yields insight into biomarkers of response and mechanisms of resistance to immune checkpoint blockade. Cancer Discovery (2016) 6(8):827–37. doi: 10.1158/2159-8290.Cd-15-1545 PMC5082984 27301722

[37] Hoshida Y Brunet JP Tamayo P Golub TR Mesirov JP. Subclass mapping: identifying common subtypes in independent disease data sets. PloS One (2007) 2(11):e1195. doi: 10.1371/journal.pone.0001195 18030330 PMC2065909

[38] Engebretsen S Bohlin J. Statistical predictions with glmnet. Clin Epigenet (2019) 11(1):123. doi: 10.1186/s13148-019-0730-1 PMC6708235 31443682

[39] Xia J Gill EE Hancock RE. Networkanalyst for statistical, visual and network-based meta-analysis of gene expression data. Nat Protoc (2015) 10(6):823–44. doi: 10.1038/nprot.2015.052 25950236

[40] Geeleher P Cox N Huang RS. Prrophetic: an R package for prediction of clinical chemotherapeutic response from tumor gene expression levels. PLoS One (2014) 9(9):e107468. doi: 10.1371/journal.pone.0107468 25229481 PMC4167990

[41] Yang W Soares J Greninger P Edelman EJ Lightfoot H Forbes S. Genomics of drug sensitivity in cancer (Gdsc): A resource for therapeutic biomarker discovery in cancer cells. Nucleic Acids Res (2013) 41(Database issue):D955–61. doi: 10.1093/nar/gks1111 PMC3531057 23180760

[42] Cotto KC Wagner AH Feng YY Kiwala S Coffman AC Spies G. Dgidb 3.0: A redesign and expansion of the drug-gene interaction database. Nucleic Acids Res (2018) 46(D1):D1068–d73. doi: 10.1093/nar/gkx1143 PMC5888642 29156001

[43] Shannon P Markiel A Ozier O Baliga NS Wang JT Ramage D. Cytoscape: A software environment for integrated models of biomolecular interaction networks. Genome Res (2003) 13(11):2498–504. doi: 10.1101/gr.1239303 PMC403769 14597658

[44] Lee SY Jeong EK Ju MK Jeon HM Kim MY Kim CH. Induction of metastasis, cancer stem cell phenotype, and oncogenic metabolism in cancer cells by ionizing radiation. Mol Cancer (2017) 16(1):10. doi: 10.1186/s12943-016-0577-4 28137309 PMC5282724

[45] Liu T Pei P Shen W Hu L Yang K. Radiation-induced immunogenic cell death for cancer radioimmunotherapy. Small Methods (2023):e2201401. doi: 10.1002/smtd.202201401 36811166

[46] Niu X Chen L Li Y Hu Z He F. Ferroptosis, necroptosis, and pyroptosis in the tumor microenvironment: perspectives for immunotherapy of Sclc. Semin Cancer Biol (2022) 86(Pt 3):273–85. doi: 10.1016/j.semcancer.2022.03.009 35288298

[47] Oh DY Fong L. Cytotoxic Cd4(+) T cells in cancer: expanding the immune effector toolbox. Immunity (2021) 54(12):2701–11. doi: 10.1016/j.immuni.2021.11.015 PMC8809482 34910940

[48] Keir ME Butte MJ Freeman GJ Sharpe AH. Pd-1 and its ligands in tolerance and immunity. Annu Rev Immunol (2008) 26:677–704. doi: 10.1146/annurev.immunol.26.021607.090331 18173375 PMC10637733

[49] Cheung EC Vousden KH. The role of ros in tumour development and progression. Nat Rev Cancer (2022) 22(5):280–97. doi: 10.1038/s41568-021-00435-0 35102280

[50] Liu TI Lu TY Yang YC Chang SH Chen HH Lu IL. New combination treatment from ros-induced sensitized radiotherapy with nanophototherapeutics to fully eradicate orthotopic breast cancer and inhibit metastasis. Biomaterials (2020) 257:120229. doi: 10.1016/j.biomaterials.2020.120229 32738654

[51] Khan AQ Rashid K AlAmodi AA Agha MV Akhtar S Hakeem I. Reactive oxygen species (Ros) in cancer pathogenesis and therapy: an update on the role of ros in anticancer action of benzophenanthridine alkaloids. BioMed Pharmacother (2021) 143:112142. doi: 10.1016/j.biopha.2021.112142 34536761

[52] Ramia E Chiaravalli AM Bou Nasser Eddine F Tedeschi A Sessa F Accolla RS. Ciita-related block of hla class ii expression, upregulation of Hla class I, and heterogeneous expression of immune checkpoints in hepatocarcinomas: implications for new therapeutic approaches. Oncoimmunology (2019) 8(3):1548243. doi: 10.1080/2162402X.2018.1548243 30723578 PMC6350839

[53] Forlani G Shallak M Gatta A Shaik AKB Accolla RS. The nlr member ciita: master controller of adaptive and intrinsic immunity and unexpected tool in cancer immunotherapy. BioMed J (2023) 46(5):100631. doi: 10.1016/j.bj.2023.100631 37467968 PMC10505679

[54] Lee HJ Lee JJ Song IH Park IA Kang J Yu JH. Prognostic and predictive value of nanostring-based immune-related gene signatures in a neoadjuvant setting of triple-negative breast cancer: relationship to tumor-infiltrating lymphocytes. Breast Cancer Res Treat (2015) 151(3):619–27. doi: 10.1007/s10549-015-3438-8 26006068

[55] Chen Y Jia Y Mao M Gu Y Xu C Yang J. Plac8 promotes adriamycin resistance via blocking autophagy in breast cancer. J Cell Mol Med (2021) 25(14):6948–62. doi: 10.1111/jcmm.16706 PMC8278087 34117724

[56] Xiang P Jin S Yang Y Sheng J He Q Song Y. Infiltrating Cd4+ T cells attenuate chemotherapy sensitivity in prostate cancer via Ccl5 signaling. Prostate (2019) 79(9):1018–31. doi: 10.1002/pros.23810 PMC6594129 31018021

[57] Das C Adhikari S Bhattacharya A Chakraborty S Mondal P Yadav SS. Epigenetic-metabolic interplay in the DNA damage response and therapeutic resistance of breast cancer. Cancer Res (2023) 83(5):657–66. doi: 10.1158/0008-5472.CAN-22-3015 PMC11285093 36661847

[58] Liu YP Zheng CC Huang YN He ML Xu WW Li B. Molecular mechanisms of chemo- and radiotherapy resistance and the potential implications for cancer treatment. MedComm (2020) (2021) 2(3):315–40. doi: 10.1002/mco2.55 PMC8554658 34766149

